# Cementless short stem total hip arthroplasty in patients older than 75 years: is it feasible?

**DOI:** 10.1007/s00402-024-05425-z

**Published:** 2024-07-05

**Authors:** Matthias Luger, Matthias Holzbauer, Matthias C. Klotz, Franz Fellner, Tobias Gotterbarm

**Affiliations:** 1 Department for Orthopedics and Traumatology, Kepler University Hospital GmbH, Krankenhausstrasse 9, 4020 Linz, Austria; 2 Johannes Kepler University Linz, Altenberger Strasse 69, 4040 Linz, Austria; 3 Marienkrankenhaus Soest, Orthopedics and Trauma Surgery, Widumgasse 5, 59494 Soest, Germany; 4 Central Radiology Institute, Kepler University Hospital, 4020 Linz, Austria

**Keywords:** Total hip arthroplasty, THA, Short stem, Cementless, Anterolateral approach, Minimally-invasive

## Abstract

**Background:**

In recent years, the indication for cementless short stem total hip arthroplasty (THA) has been widened to elderly patients as they might profit by the advantages of the short-curved implant design as well. Therefore, this study was conducted to evaluate the clinical and radiological outcome of a cementless short stem in elderly patients (≥ 75 years) compared to a young control group (≤ 60 years).

**Methods:**

A retrospective cohort of 316 THAs performed between 2014 and 2017 was prospectively examined. In all patients a cementless, curved short stem and press-fit cup (Fitmore® stem; Allofit®/-S cup; both ZimmerBiomet, Warsaw, IN, USA) were implanted via a minimally-invasive anterolateral approach. Clinical and radiological outcome as well as rate of complications and revision were assessed.

**Results:**

In total, 292 patients have been included for analysis of complications and revisions (Øfollow-up: 4.5 years) and 208 patients for clinical and radiological outcome (Øfollow-up: 4.4 years). Complication rate was significantly increased in elderly patients (13.7% vs. 5.8%, p = 0.023), while the revision rate was increased without statistical significance (5.2% vs. 2.2%, p = 0.169). Periprosthetic fractures occurred significantly higher in the elderly patients (5.2% vs. 0.7%; p = 0.026). Both groups showed a comparable clinical outcome in the Harris Hip Score (93.7 vs. 91.9; p = 0.224), Oxford Hip Score (44.5 vs. 43.7; p = 0.350), Forgotten Joint Score (81.7 vs. 81.5; p = 0.952) and WOMAC (7.4 vs. 9.3; p = 0.334).

**Conclusion:**

Cementless short stem total hip arthroplasty shows a comparable clinical and radiological outcome in patients over 75 years of age compared to younger patients under 60 years of age. However, cementless shorts stem THA shows an increased rate of overall complications and periprosthetic fractures in elderly patients over 75 years of age. Cemented fixation of the femoral component should be considered in patients over 75 years of age.

**Level of evidence:**

III Case-controlled study.

**Trial registration:**

Observational study without need for trial registration due to ICMJE criteria.

## Introduction

Cementless short stems have proven to show excellent survival rates ranging from 95 to 100% after 3–11 years [1–5]. Short stems have been increasingly used in total hip arthroplasty (THA) in parallel with minimally invasive (MIS) approaches as they facilitate soft tissue sparing implantation [6, 7]. Additionally, they preserve more of the proximal femoral bone stock and can restore the proximal femoral anatomy more accurately [7–9]. Short stems can even reduce the rate of periprosthetic fractures in MIS approaches compared to conventional cementless straight stems [10, 11].

Because of its bone preserving quality, the use of short stems in THA has initially been recommended for young and active patients with adequate bone quality [3]. In recent years, the indication for cementless short stem THA has been widened also in geriatric patients, as this patient collective might also profit by the theoretical advantages of short stem THA [12–14]. In a multicenter case study with 400 patients, Gkagkalis et al. [12] report a comparable clinical outcome and complication rate in patients with an advanced age over 75 years, when using a neck-sparing cementless short stem. However, they advocate against the use of this type of stem in patients with a Dorr type C of the proximal femur due the significantly increased risk of periprosthetic fractures [12].

The use of cementless fixation and cementless short stems in elderly patients contradicts registry data [15–17]. Periprosthetic femoral fractures and the safest implant fixation remain the most important aspects in THA in patients older than 75 years, in order to reduce the revision risk and increase the long-term survival of primary THAs [15–17].

As data on the performance of cementless short stems in elderly patients is still rare, this study was conducted to compare the survival, complication and revision rate as well as clinical and radiological outcome of a neck-resecting cementless short stem in elderly patients over 75 years compared to a younger control group with patients under 60 years of age.

## Methods

### Study design

A retrospective cohort of 316 short stem THAs performed between 2014 and 2017 was prospectively examined in a comparative study. The study was approved by the institutional review board (1239/2019) and conducted according to the Helsinki Declaration of 2008. Informed consent was obtained in every case for participation in the study.

### Cohort

A cohort of 316 short stem THAs in 300 patients was analyzed for inclusion. Two age groups were included for the analysis of clinical and radiological follow-up, as well as of complications and rate of revision. One age group with patients under 60 years of age at index surgery and one group of patients older than 75 years of age at index surgery were included in the study. The younger patients were included as the control group and the elderly patients were included as the treatment group. In the younger control group 149 THAs in 140 patients and in the elderly treatment group 167 THAs in 160 patients were performed between 2014 and 2017. Indication for surgery were primary osteoarthritis, avascular necrosis of the femoral head, congenital dysplasia of the hip (Crowe grade I), secondary osteoarthritis such as posttraumatic conditions as well as rheumatoid arthritis. Patients with bilateral THA or prior hip surgery have also been included.

All surgeries were performed at a single tertiary university hospital. Patients were followed at the recommended follow-up examinations. A minimum follow-up of 2 years was required for inclusion. The routine interval for a follow-up examination at the study center are at 3 months, 1, 3, 5, 7 and 10 years postoperatively. If a patient missed a follow-up examination, the patient was invited for a clinical and radiological follow-up examination. If the patient refused to participate personally, the patient was asked for any complication, revision surgery or reoperation within the last follow-up examination given the informed consent. If a patient was deceased, any complication, revision surgery or reoperation was excluded using information from relatives, general practitioners and clinical records. Therefore, the both study groups were reviewed in one collective for complications and revisions as well as one collective for clinical and radiological outcome.

### Implants & surgery

A cementless, curved short stem (Fitmore® stem, ZimmerBiomet, Warsaw, IN, USA) and a cementless titanium press-fit cup with or without screws (Allofit®/-S, ZimmerBiomet, Warsaw, IN, USA) was implanted in every case. Fitmore® hip stem is a titanium alloy stem (Ti Al6V4) that has a porolock Ti-VPS coating in the proximal part to enhance bone ingrowth and is available in four different neck angle options (127°, 129°, 137°, 140°). A highly cross-linked polyethylene liner (Alpha Durasul®, ZimmerBiomet, Warsaw, IN, USA) and a ceramic femoral head (BIOLOX forte, CeramTec GmbH, DE; Sulox, ZimmerBiomet, Warsaw, IN, USA) were used. Digital templating was conducted in every case using mediCAD® version 5.1 (Hectec GmbH, Altdorf, Germany).

In total, 13 surgeons performed the surgeries. The surgeries were performed by 6 consultants and 4 residents. 3 surgeons performed the surgeries as residents and after finishing residency also as consultants. Consultants performed at least 50 arthroplasties per year at the institution. Surgeries performed by residents were always conducted under supervision of a consultant.

All surgeries were performed under laminar air flow. Extremity preparation was performed with threefold antiseptic scrub with alcohol disinfectant in all cases. The standardized peri- and postoperative protocol was identical in all cases, including single-shot antibiotics [cefuroxime 1.5 g intravenous (i.v.), directly preoperatively], full weight-bearing as tolerated from the first postoperative day on, indomethacin 75 mg twice daily for the prevention of heterotopic ossification on days 1–4 postoperatively, and 40 mg low-molecular-weight heparin or rivaroxaban 10 mg for 28 days postoperatively as venous thromboembolic event prophylaxis. Suturing was done either by intracutaneous suturing. Fluoroscopy was not routinely used.

A minimally invasive anterolateral approach was performed in supine positioning [18]. A skin incision was centered over the greater trochanter. An incision at the border between the Tensor fasciae latae and the Tractus iliotibilias was performed. Then, the Watson-Jones interval between Tensor fasciae latae and Gluteus medius was bluntly dissected. A capsulectomy was performed in each case. The average operation time from skin incision to closure was 78.3 min (min.: 48,1; max.: 200 min).

### Assessment of complications and revisions

All complications and revisions have been analyzed through the follow-up appointments, retrospectively via the electronic institutional database as well as prospectively via consulting all patients. The type of complication was recorded. Complications were defined as a periprosthetic fracture (PFF), periprosthetic joint infection (PJI), nerve lesion (confirmed by electroneurography), wound healing disorder, and hematoma or seroma that lead to an intervention, a prolonged hospital stay, an unplanned follow-up visit or readmission. Implant loosening was defined by the criteria of Engh et al. [19]. PFFs were analyzed depending on the intra- or postoperative occurrence, the classification according to Dorr et al. [20], the fracture type according to the Vancouver Classification [21, 22] and the time of occurrence. Revision was defined as a change of modular parts or removal of the components. Reoperation was defined as an operation without the change of components.

### Clinical and radiographic assessment

Clinical follow-up was conducted through postoperative measurements of functional outcome, patient report outcome measurements (PROMs) and assessment of quality of life. At the follow-up every patient was assessed with the Harris hip score (HHS) [23], the Oxford hip score (OHS) [24], the Forgotten Joint Score (FJS) [25] and the Western Ontario and McMaster Universities Arthritis Index (WOMAC) [26]. The sports activity was measured by using the University of California Los Angeles (UCLA) activity score [27]. Quality of life was assessed by using the European Quality of Life 5 Dimension 3 Level (EQ-5D-3L) [28] and the Veterans RAND 12-Item Health Survey (VR-12) [29]. Pain at rest was measured with the visual analog scale (VAS) with a scale of 0–10 [30].

Radiological follow-up was conducted through standardized digital, calibrated low centered anterior–posterior radiograph of the pelvis and a radiograph of the lateral hip [31]. Radiological outcome was assessed for heterotopic ossifications, cortical hypertrophies, radiolucent lines, bone resorptions and osteolysis. Heterotopic ossifications were assessed according to the Brooker-classification [32]. Cortical hypertrophies and radiolucent lines were assessed as previously described [1, 5]. Bone resorptions were evaluated according to the Singh-Index [33]. A Singh-Index of 1–3 was defined as a bone resorption [12, 33]. Cortical hypertrophies, radiolucent lines, bone resorptions and osteolysis were evaluated using the zones described by Gruen et al. [34]. Implant loosening as defined by the criteria of Engh et al. [19].

### Statistical analysis

Descriptive analyses were performed for patient demographics. A Shapiro–Wilk test for normality was performed to determine whether continuous data were normally distributed. As the variables were normally distributed, Pearson’s chi square tests were performed for categorial variables and student’s t-tests were performed for continuous variables. Values are given as mean values with standard deviation. The endpoint of stem survival was measured for revision for any reason and for stem revision by using a Kaplan–Meier survival analysis with a 95% confidence interval (CI). A p-value of < 0.05 was considered to be significant. Data was analyzed using SPSS version 28 (IBM SPSS statistics, Chicago, IL) and R version 4.3.3 (R Development Core Team).

## Results

**Fig. 1:**
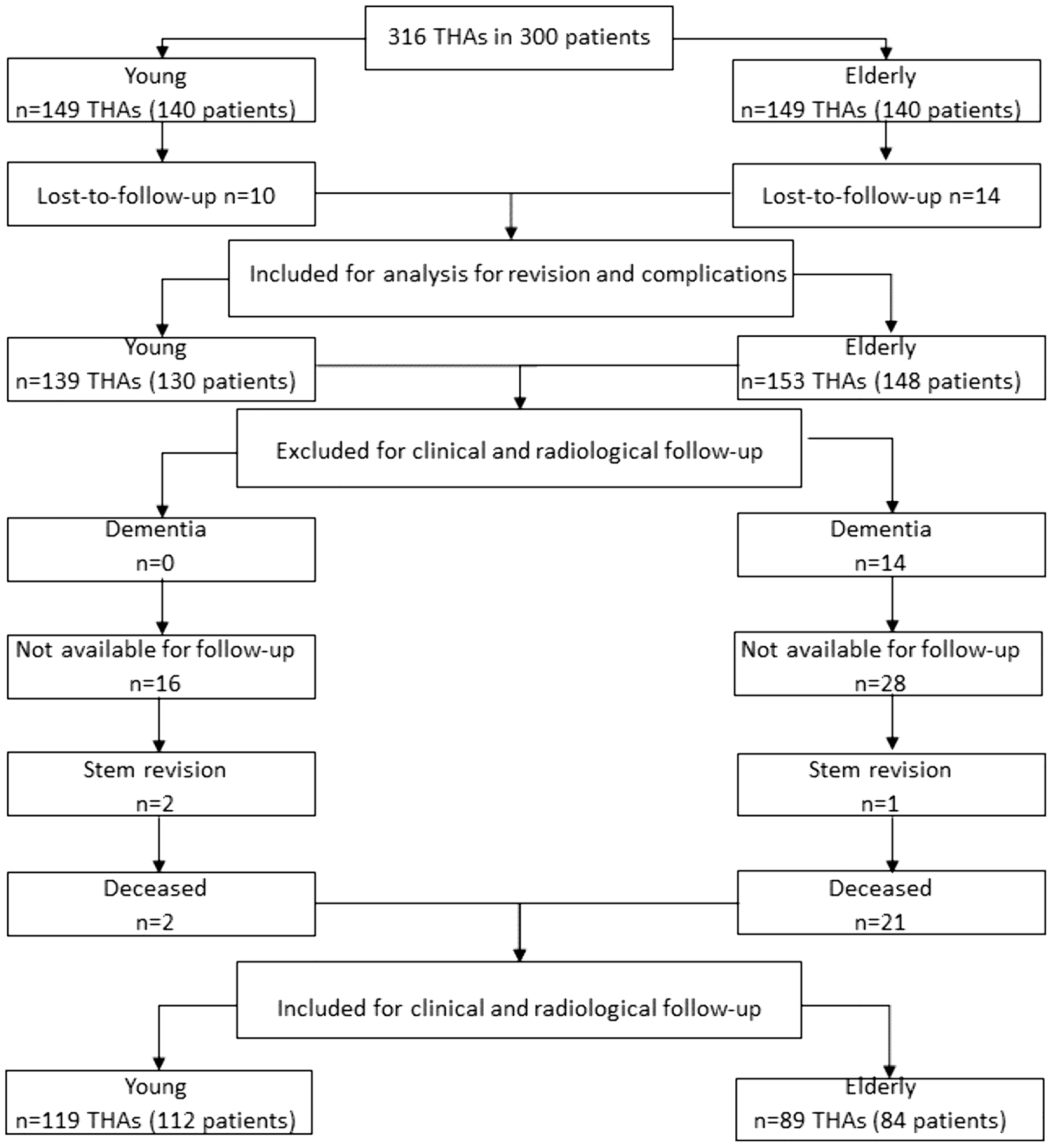
Consort diagram for inclusion and exclusion

In total, 292 patients (92.4%) were included for analysis of complications and revisions with a conclusive follow-up. For clinical and radiological outcome 208 patients (65.8%) were included in the study (Fig. 1). In the younger group 10 patients (3.2%) and in the elderly group 14 patients (4.4%) were lost-to-follow-up. In the elderly group 14 patients (4.4%) were not available for assessment of the questionnaires due to dementia and 28 patients (8.9%) refused to attend the clinical or radiological follow-up examination due to their advanced age, personal reasons or the COVID-restrictions at that time. One patient (0.3%) was excluded due to stem exchange to a straight stem and therefore not included for the final analysis of clinical and radiological outcome. Furthermore, 21 patients (6.6%) deceased without any revision surgery until death. In the younger group, 2 patients (0.6%) deceased without any revision surgery and 2 patients (0.6%) were excluded for clinical and radiological follow-up due to stem revision. Furthermore, 16 patients (5.1%) refused to attend the clinical or radiological follow-up examination due to personal reasons or the COVID-restrictions at that time.

### Analysis for complications and revisions

**Fig. 2:**
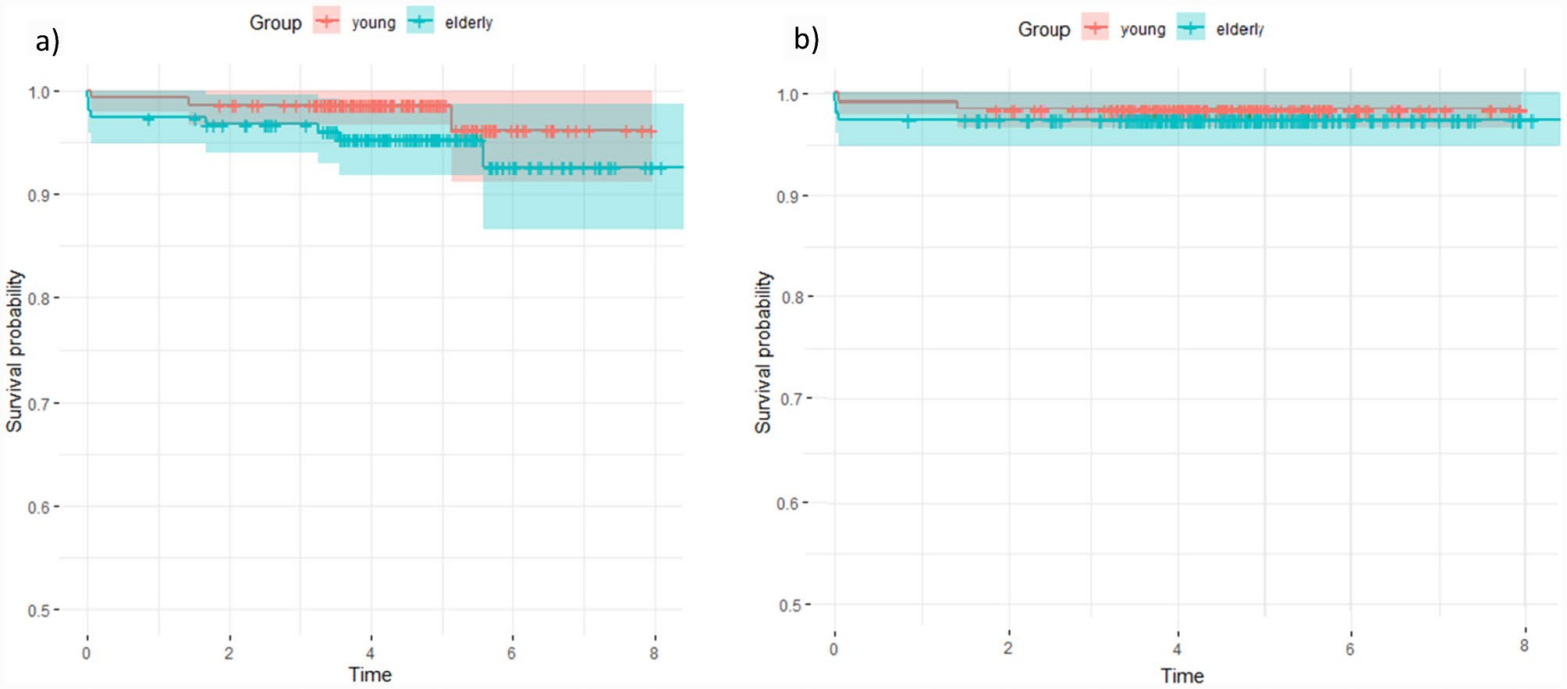
**a**, **b**: Kaplan Meier survival after 8.6 years for the endpoint: **A** “revision for any reason” and **B** “stem revision” for young and elderly patients (n = 292)

**Fig. 3:**
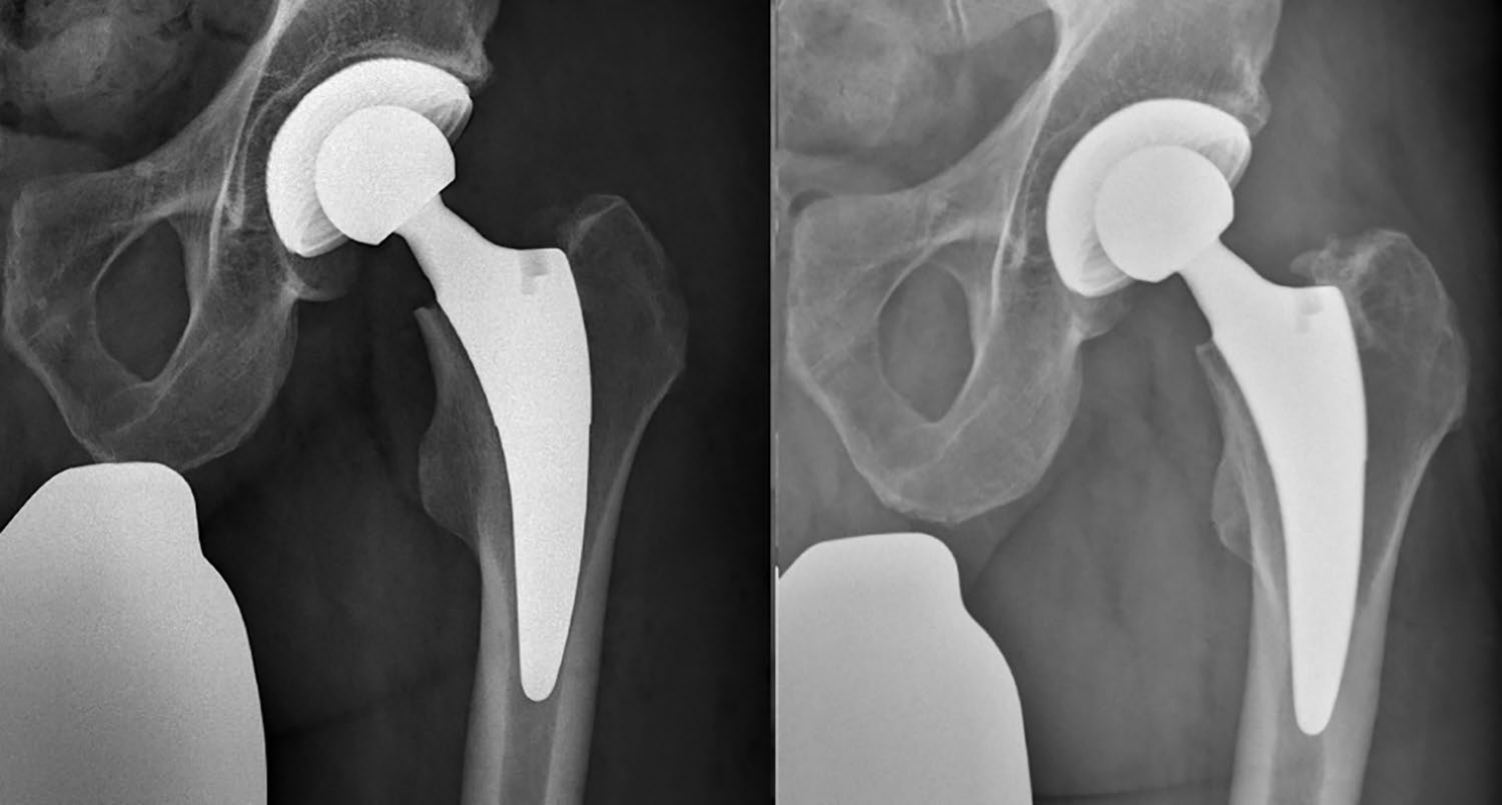
49-year-old-male patient; left: fourth postoperative day; right: at 4.3 years of follow-up

**Fig. 4:**
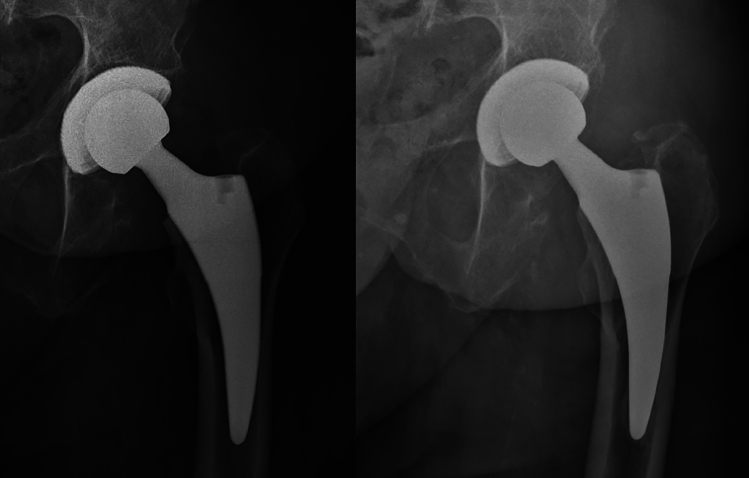
79-year-old female; left: fourth postoperative day; right: at 5.2 years of follow-up

**Fig. 5:**
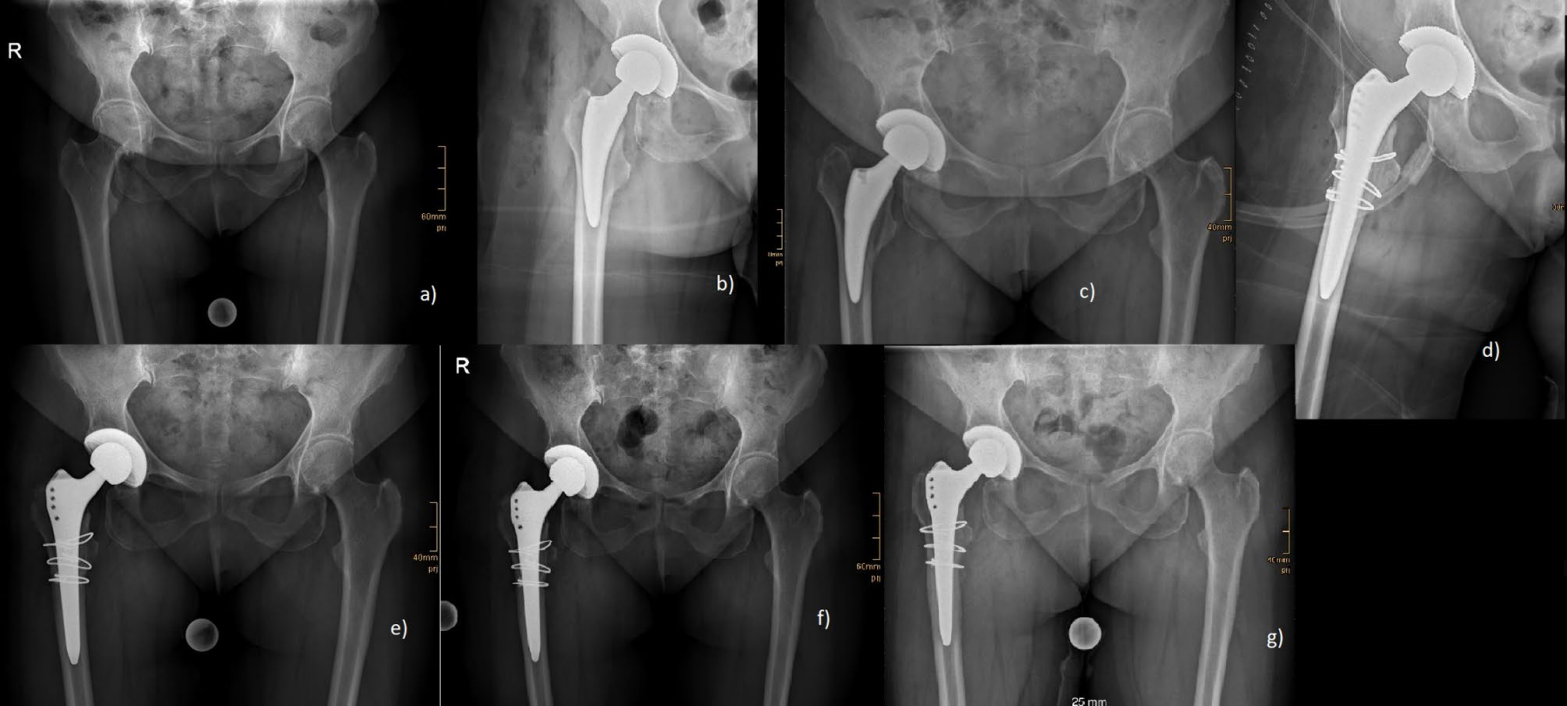
Occult fracture of the medial cortex detected on the fourth postoperative day; **a** preoperative; **b** postoperative; **c** fourth postoperative day; **d** postoperatively after revision (Alloclassic SL (ZimmerBiomet®) with three cerclage wires); **e** 6 weeks after revision; **f** 3 months after revision; **g** 1 year after revision

**Fig. 6:**
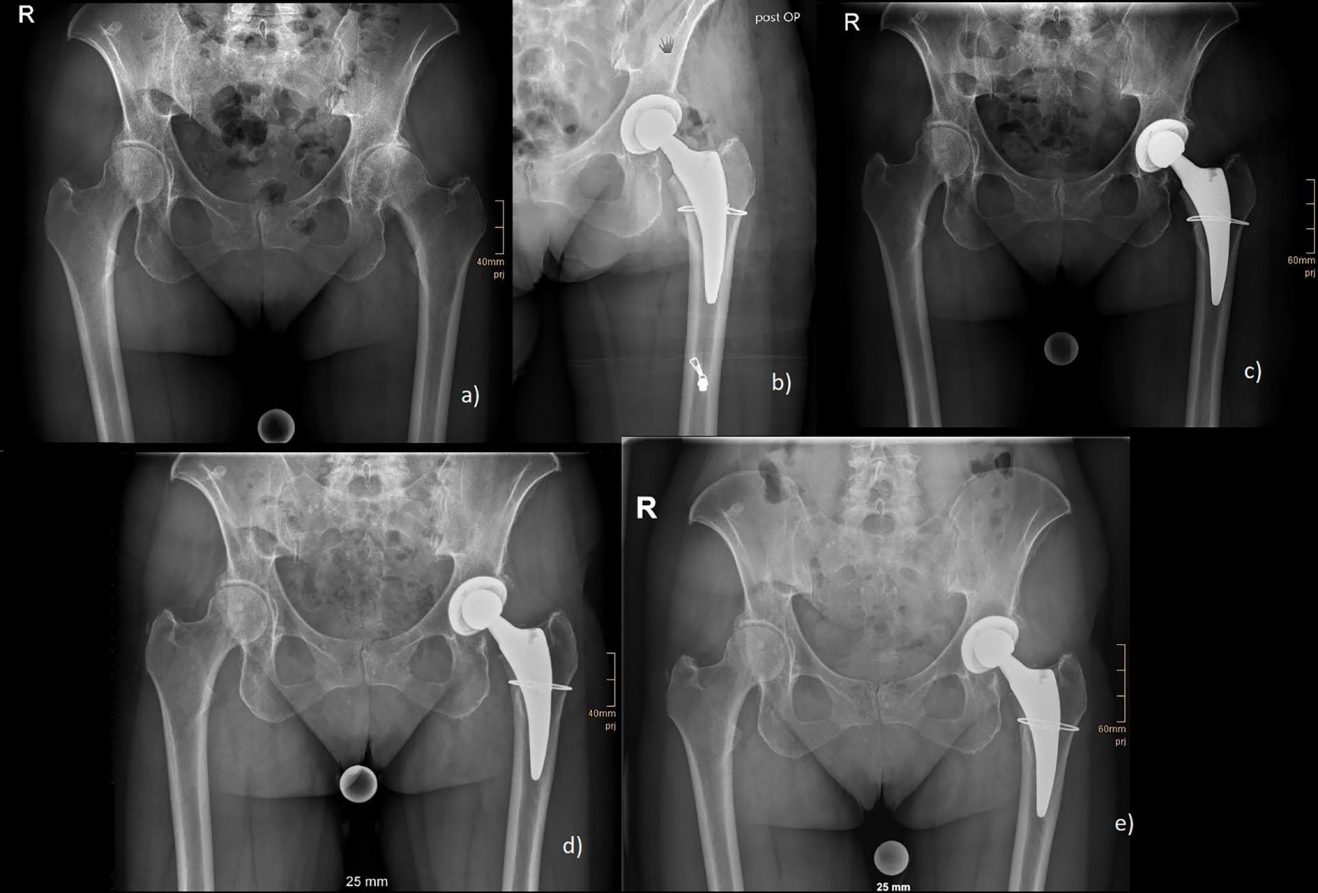
Intraoperative fracture of the calcar treated with one cerclage wire: **a** preoperative; **b** postoperative; **c** 6 weeks postoperative; **d** 3 months postoperative; **e** 1 year postoperative

The patient demographics for both groups for the analysis of complications and revisions are given in Table 1. Both groups differed significantly in age at surgery (p < 0.001), gender (p = 0.032), the indication for surgery (p < 0.001), ASA score (< 0.001) and the Dorr type (p = 0.014). The rates of complications and revisions are given in detail in Table 2. Overall complication rate was significantly higher in the elderly patients (13.7% vs. 5.8%, p = 0.023), (Table 2). Periprosthetic fractures also occurred significantly higher in the elderly patients (5.2% vs. 0.7%; p = 0.026). Analysis of the occurrence of PFFs depending on specific factors did not show any statistically significant difference, (Table 2). All other complications were also without any significant difference between both groups (Table 2). Rates for revision for any reason, as well as stem revision were higher in the elderly group, but without statistically significant difference (p = 0.169; p = 0.479) (Tab. 2). Figure 2 shows the Kaplan Meier survival analysis after 8.6 years. Figure 2a shows the survival analysis for the endpoint “revision for any reason” with 97.8% (CI: 91.1–100%) for young patients and 94.8% (CI: 86.5–98.7%) for elderly patients. Figure 2b shows the survival analysis for the endpoint “stem revision” with 98.6% (CI: 96.6–100%) for young patients and 97.4% (CI: 94.9–99.9%) for elderly patients. Radiographic images of a standard case in the younger age group are given in Fig. 3 and in the elderly age group in Fig. 4. Examples of cases with PFFs are shown in Figs. 5 and 6.

**Table 1:** Patient demographics for analysis of complications and revisions

	Young < 60 years of age	Elderly > 75 years of age	P Value
Total number of hips	139 (130 patients)	153 (148 patients)	
Gender			**0.032**
Woman	68 (48.9%)	94 (61.4%)	
Men	71 (51.1%)	59 (38.6%)	
Age at Surgery (in years)	52.4 ± 6.2	79.8 ± 3.9	** < 0.001**
Indication for surgery			** < 0.001**
Primary osteoarthritis	109 (78.4%)	129 (84.3%)	
Avascular necrosis of the femoral head	12 (8.6%)	22 (14.4%)	
Congenital dysplasia of the hip	17 (12.2%)	2 (1.3%)	
Secondary osteoarthritis	1 (0.7%)	0 (0.0%)	
Side			0.750
Left	68 (48.9%)	72 (47.1%)	
Right	71 (51.1%)	81 (52.9%)	
ASA Score			** < 0.001**
1	58 (41.7%)	9 (5.9%)	
2	70 (50.4%)	79 (51.6%)	
3	11 (7.9%)	65 (42.5%)	
4	0 (0.0%)	0 (0.0%)	
BMI	27.6 ± 5	27.2 ± 4	0.448
Surgeon’s experience			0.314
Consultant	103 (74.1%)	121 (79.1%)	
Resident	36 (25.9%)	32 (20.9%)	
Dorr Classification			**0.014**
A	42 (30.2%)	27 (17.6%)	
B	81 (58.3%)	95 (62.1%)	
C	16 (11.5%)	31 (20.3%)	

Bold letters indicate significant values

*ASA* Score American Society of Anesthesiologists Score; *BMI* Body Mass Index, kg/m^2^

**Table 2:** Complications and revisions

	Young < 60 years of age	Elderly > 75 years of age	P Value
Average Follow-up (in years)	4.5	4.6	0.571
Min.-Max. (in years)	0.05–8	0–8.6	
Complication			**0.023**
Yes (%)	8 (5.8%)	21 (13.7%)	
No (%)	131 (94.2%)	132 (86.3%)	
Periprosthetic Fracture			**0.026**
Yes (%)	1 (0.7%)	8 (5.2%)	
No (%)	138 (99.3%)	146 (95.4%)	
Intraoperative	0 (0.0%)	3 (2.0%)	0.097
Vancouver A	0 (0.0%)	2 (1.3%)	0.176
Vancouver B	0 (0.0%)	1 (0.7%)	0.340
Vancouver C	0 (0.0%)	0 (0.0%)	-
Postoperative	1 (0.7%)	5 (3.3%)	0.125
Vancouver A	0 (0.0%)	2 (1.3%)	0.176
Vancouver B	1 (0.7%)	3 (2.0%)	0.362
Vancouver C	0 (0.0%)	0 (0.0%)	-
Time of occurrence (months)	0 ± 0.0	21.3 ± 27.5	0.496
< 12 months	1 (0.7%)	5 (3.3%)	0.125
> 12 months	0 (0.0%)	3 (2.0%)	0.097
Dorr Classification			
A	0 (0.0%)	3 (2.0%)	0.097
B	1 (0.7%)	3 (2.0%)	0.362
C	0 (0.0%)	2 (1.3%)	0.176
Sex			-
Female	1 (0.7%)	4 (4.3%)	0.811
Male	0 (0.0%)	3 (5.1%)	
Periprosthetic Joint Infection	0 (0.0%)	3 (2.0%)	0.097
Aseptic loosening	1 (0.7%)	0 (0.0%)	0.293
Dislocation	1 (0.7%)	1 (0.7%)	0.946
Femoral Nerve lesion	0 (0.0%)	1 (0.7%)	0.340
Wound healing disorder	2 (1.4%)	5 (3.3%)	0.307
Hematoma/seroma	1 (0.7%)	3 (2.0%)	0.362
Revision			
Revision for any reason	3 (2.2%)	8 (5.2%)	0.169
Stem Revision	2 (1.4%)	4 (2.6%)	0.479
Reoperation	1 (0.7%)	0 (0.0%)	0.293

Bold letters indicate significant values

### Clinical and radiological outcome

For clinical and radiological outcome 89 THAs have been included in the elderly group and 119 THAs in the younger group with an average follow-up of 4.4 years in both groups (p = 0.933). The patient demographics for both groups are given in Table 3. Both groups differed significantly in age at surgery (p < 0.001), the indication for surgery (p = 0.013), ASA score (< 0.001) and the Dorr type (p = 0.049). Expectedly, the number of congenital dysplasia of the hip was higher in the younger group (12.6% vs. 1.1%), as well as the number of patients with ASA score 1 (41.2% vs. 7.9%). The number of patients with ASA score 3 was higher in the elderly group (36% vs. 7.6%), as well as patients with a Dorr type C (19.1% vs. 12.6%).

**Table 3:** Patient demographics for analysis of clinical and radiological outcome

	Young < 60 years of age	Elderly > 75 years of age	P Value
Total number of hips	119 (112 patients)	89 (84 patients)	
Gender			0.112
Woman	58	53	
Men	61	36	
Age at Surgery (in years)	51.8 ± 6.4	79.1 ± 3.3	** < 0.001**
Indication for surgery			**0.013**
Primary osteoarthritis	93 (78.2%)	77 (86.5%)	
Avascular necrosis of the femoral head	10 (8.4%)	11 (12.4%)	
Congenital dysplasia of the hip	15 (12.6%)	1 (1.1%)	
Secondary osteoarthritis	1 (0.8%)	0 (0.0%)	
Side			0.643
Left	56 (47.1%)	39 (43.8%)	
Right	63 (52.9%)	50 (56.2%)	
ASA Score			** < 0.001**
1	49 (41.2%)	7 (7.9%)	
2	61 (51.3%)	50 (56.2%)	
3	9 (7.6%)	32 (36%)	
4	0 (0.0%)	0 (0.0%)	
BMI	27.5 ± 5.2	27 ± 3.5	0.360
Surgeon’s experience			0.101
Consultant	86 (72.3%)	73 (82%)	
Resident	33 (27.7%)	16 (18%)	
Dorr Classification			**0.049**
A	37 (31.1%)	15 (16.9%)	
B	67 (56.3%)	57 (64%)	
C	15 (12.6%)	17 (19.1%)	

Bold letters indicate significant values

*ASA Score* American Society of Anesthesiologists Score; *BMI* Body Mass Index, kg/m^2^

The detailed results for the clinical outcome are given in Table 4. Clinical outcome did not differ between both groups for the HHS (p = 0.224), OHS (p = 0.350), FJS (p = 0.952) and WOMAC (p = 0.334) (Tab. 4). The average score of the VAS at rest was also without any statistically significant difference (p = 0.102) (Table 4). Both groups differed significantly in the UCLA with a higher activity level in the younger age group (6.9 vs. 4.6, p < 0.001). The quality of life was significantly higher for younger patients in the EQ-5D-3L (p = 0.003) (Table 4). The physical component summary (PCS) of the VR-12 questionnaire was also significantly higher for younger patients (p < 0.001), whereas the mental component summary (MCS) of the VR-12 was without any significant difference between both age groups (p = 0.238) (Table 4).

**Table 4:** Clinical outcome; Bold letters indicate significant values

	Young < 60 years of age	Elderly > 75 years of age	P Value
Average Follow-up (in years)	4.4 ± 1	4.4 ± 1	0.933
Min.-Max. (in years)	2.1–7.1	2–7.1	
Pain			0.146
Yes (%)	42 (35.3%)	23 (25.8%)	
No (%)	77 (64.7%)	76 (74.2%)	
VAS (± SD)	.85 ± 1.4	.56 ± 1.1	0.102
Harris Hip Score (± SD)	93.7 ± 11.2	91.9 ± 8.8	0.224
Oxford Hip Score (± SD)	44.5 ± 7.2	43.7 ± 5.3	0.350
Forgotten Joint Score 12 (± SD)	81.7 ± 26.3	81.5 ± 22	0.952
WOMAC Score (± SD)	7.4 ± 14.8	9.3 ± 12.3	0.334
UCLA Score (± SD)	6.9 ± 2.3	4.6 ± 2.3	** < 0.001**
EQ-5D-3L (%)	82.3 ± 19	75.9 ± 11.9	**0.003**
EQ-5D-3L Q1 (median)	2.4 (1)	1.3 (1)	0.160
EQ-5D-3L Q2 (median)	1.1 (1)	1.2 (1)	** < 0.001**
EQ-5D-3L Q3 (median)	1.1 (1)	1.4 (1)	** < 0.001**
EQ-5D-3L Q4 (median)	1.4 (1)	1.6 (2)	**0.002**
EQ-5D-3L Q5 (median)	1.1 (1)	1.2 (1)	0.075
VR-12 PCS (± SD)	54.7 ± 10.9	47.9 ± 10.2	** < 0.001**
VR-12 MCS (± SD)	43.4 ± 4.3	44.3 ± 5.8	0.238

The detailed results for the radiological outcome are given in Table 5. Heterotopic ossifications were slightly higher in the younger age group (16% vs. 10.1%, p = 0.221) without any statistical significance, also when assessed according to the Brooker-classification (p = 0.088) (Table 5). Cortical hypertrophies were significantly higher in the younger age group (54.6% vs. 31.5%, p < 0.001) (Table 5). Cortical hypertrophies were detected in the Gruen zones 3 and 5 with statistically significantly higher proportion in the younger age group (P < 0.001; p = 0.002) (Table 5). Radiolucent lines were significantly higher in younger patients (13.4% vs. 2.2%, p = 0.004), especially in Gruen zone 1 (p = 0.008) (Table 5). Bone resorptions have been detected without any statistical significance between groups (p = 0.658) (Table 5). Osteolysis was not detected in any age group.

**Table 5:** Radiological outcome

	Young < 60 years of age	Elderly > 75 years of age	P Value
Average Follow-up (in years)	4.4 ± 1	4.4 ± 1	0.933
Min.-Max. (in years)	2.1–7.1	2–7.1	
Heterotopic ossifications			0.221
Yes (%)	19 (16%)	9 (10.1%)	
No (%)	100 (84%)	80 (89.9%)	
Brooker-Classification			0.088
0	100 (84%)	80 (89.9%)	
1	16 (13.5%)	7 (7.9%)	
2	3 (2.5%)	0 (0.0%)	
3	0 (0.0%)	2 (2.2%)	
Cortical hypertrophy			** < 0.001**
Yes (%)	65 (54.6%)	28 (31.5%)	
No (%)	54 (45.4%)	61 (68.5%)	
Gruen zone 1	0 (0.0%)	0 (0.0%)	–
Gruen zone 2	0 (0.0%)	0 (0.0%)	–
Gruen zone 3	64 (53.8%)	27 (30.3%)	** < 0.001**
Gruen zone 4	0 (0.0%)	0 (0.0%)	–
Gruen zone 5	37 (31.1%)	11 (12.4%)	**0.002**
Gruen zone 6	0 (0.0%)	0 (0.0%)	–
Gruen zone 7	0 (0.0%)	0 (0.0%)	–
Radiolucent Line (< 2mm)			**0.004**
Yes (%)	16 (13.4%)	2 (2.2%)	
No (%)	103 (86.6%)	87 (97.8%)	
Gruen zone 1	9 (7.6%)	0 (0.0%)	**0.008**
Gruen zone 2	1 (0.8%)	0 (0.0%)	0.386
Gruen zone 3	3 (2.5%)	0 (0.0%)	0.131
Gruen zone 4	3 (2.5%)	2 (2.2%)	0.898
Gruen zone 5	2 (1.7%)	0 (0.0%)	0.219
Gruen zone 6	0 (0.0%)	0 (0.0%)	–
Gruen zone 7	2 (1.7%)	0 (0.0%)	0.219
Bone resorption			0.658
Yes (%)	7 (5.9%)	4 (4.5%)	
No (%)	112 (94.1%)	85 (95.5%)	
Gruen zone 1	0 (0.0%)	0 (0.0%)	–
Gruen zone 2	0 (0.0%)	0 (0.0%)	–
Gruen zone 3	0 (0.0%)	0 (0.0%)	–
Gruen zone 4	0 (0.0%)	0 (0.0%)	–
Gruen zone 5	0 (0.0%)	0 (0.0%)	–
Gruen zone 6	0 (0.0%)	0 (0.0%)	–
Gruen zone 7	7 (5.9%)	4 (4.5%)	0.658
Osteolysis			–
Yes (%)	0.0 (0%)	0.0 (0%)	
No (%)	100 (100%)	100 (100%)	

Bold letters indicate significant values

## Discussion

This study reports the clinical and radiological outcome, as well as the rate of complications and revisions in cementless short stem total hip arthroplasty in elderly patients over 75 years of age compared to a young control group under 60 years of age. While clinical outcome and patient satisfaction is comparable in both outcome groups, elderly patients show a higher complication and revision rate.

The clinical and radiological outcome in the study is comparable to other studies of the Fitmore® hip stem. The clinical outcome is comparable for both age groups without any significant difference in the HHS, OHS, FJS-12 as well as WOMAC Score. Our findings are comparable to other studies evaluated the clinical outcome and PROMs after short stem THA [1, 12, 35, 36]. As to be expected, our results show a higher activity level of younger patients with a significantly higher UCLA score as well as PCS-12 of the VR-12 questionnaire. Additionally, the quality of life is significantly lower for elderly patients in the EQ-5D-3L, while the mental health is comparable without any significant difference in the MCS-12 of the VR-12 questionnaire.

The Fitmore® stem shows good to excellent early outcomes [6, 37] with reliable fixation in a radiostereometric analysis 2 years after surgery [37], as well as in Ein-Bild-Roentgen-Analysis Femoral-Component-Analysis (EBRA-FCA) 5 years after surgery [38]. Innmann et al. [5] report a low revision rate of 93.7% for all stem revisions and 99.6% for revision due to aseptic loosening after a follow-up of 8.6 years. Our study finds a comparable revision rates for young and elderly patients with the same stem design. The higher revision rate for revisions for any reason might also be associated with the generally higher age and might not be associated with the stem design and related complications.

In numerous studies cementless fixation in elderly patients is not advocated due to the increased risk for periprosthetic femoral fractures [15–17, 39]. In recent studies, cementless short stems show a low number of PFFs and they are associated with a reduced risk compared to standard cementless straight stems [10, 11, 40]. Gkagkalis et al.[12] do not report an overall increased fracture rate for the Optimys® short stem (Mathys, Bettlach, CH) with 1.5% in patients under 60 years and 1.4% in patients over 75 years of age at index surgery. However, they advocate against the use of this type of short stem in patients with a Dorr type C, as PFFs occurred in 22.2% of all patients with a Dorr type C femur [12]. The rate of PFFs was significantly increased in the presented study for elderly patients with 5.2% compared to 0.7% in young patients (p = 0.026). However, our results do not show a statistical significance for intra- or postoperative occurrence, early or late occurrence, as well as the Dorr type. In a big propensity-score-matched analysis, increased age was found to be a risk factor for the occurrence of a PFFs within the first year for the Fitmore® stem [11]. The results in the presented study supports this finding with a significantly increased overall fracture rate in elderly patients without any significant difference in the subgroup analysis. The higher fracture rate might be associated with the cementless fixation itself, as cementless short stems show a generally low number of PFFs [10–12, 40].

The Fitmore® hip stem is associated with a high number of cortical hypertrophies [1, 5, 41]. CHs are reported up to 56% for this stem design and was not associated with inferior clinical results after 3.3 years and up to 8.6 years after index surgery [1, 5, 37]. The radiological outcome in the presented study shows a significantly increased rate of CHs within the younger age group of 54.6% compared to 31.5% in elderly patients (p < 0.001), primarily determined in the Gruen zones 3 and 5 comparable to other studies [1, 5, 37]. Our results show a higher activity level in the patients under 60 years of age with a statistically higher UCLA score as well as the PCS-12 of the VR-12 questionnaire. The higher activity level and therefore higher pressure on the cortical bone might lead to a higher number of CHs. Other comparable stem designs show high a rate of aseptic loosening in young and active patients under 60 years of age after a follow-up of five years [42], which could not be seen in the presented study, as we report only one case of aseptic loosening within an average clinical follow-up of 4.4 years.

Limitations of the study are primarily the retrospective study cohort. Therefore, we cannot present preoperative clinical scores and PROMs. Furthermore, there has not been any randomized controlled study concept. A low number of patients lost-to-follow-up can be presented in the study in respect of the follow-up for revision. However, a significant number of patients did not participate in the clinical and radiological follow-up due to several reasons, especially the COVID-restrictions at the time of the follow-up examinations.

## Conclusion

Cementless short stem total hip arthroplasty shows a comparable clinical and radiological outcome in patients over 75 years of age compared to younger patients under 60 years of age. However, cementless shorts stem THA shows an increased rate of overall complications and periprosthetic fractures in elderly patients over 75 years of age. Cemented fixation of the femoral component should be considered in patients over 75 years of age.

## Data Availability

Data and materials are available on request.
